# Effects of ABCA1 R219K Polymorphism and Serum Lipid Profiles on Mild Cognitive Impairment in Type 2 Diabetes Mellitus

**DOI:** 10.3389/fnagi.2017.00257

**Published:** 2017-08-03

**Authors:** Rongrong Cai, Jing Han, Jie Sun, Rong Huang, Sai Tian, Yanjue Shen, Shaohua Wang

**Affiliations:** ^1^Department of Endocrinology, The Affiliated ZhongDa Hospital of Southeast University Nanjing, China; ^2^Medical school of Southeast University Nanjing, China

**Keywords:** high density lipoprotein cholesterol, ATP-binding cassette A1 transporter, type 2 diabetes mellitus, mild cognitive impairment, memory

## Abstract

**Background:** Accumulated evidence suggests that adverse lipid changes are risk factors for type 2 diabetes mellitus (T2DM) and neurodegenerative disorders. The ATP-binding cassette A1 transporter (ABCA1) gene contributes to both lipid processing and amyloid-β formation and thus shows promise as a biological target in the pathology of mild cognitive impairment (MCI) in T2DM.

**Objective:** This study aimed to investigate the interactions among lipids, ABCA1 R219K polymorphism, and cognitive function in T2DM.

**Methods:** Clinical parameters, including lipids, were measured. The testing scores of different cognitive domains were recorded, and the ABCA1 R219K polymorphisms were analyzed.

**Results:** A total of 226 patients, including 124 MCI patients and 102 controls, were enrolled in this study. T2DM patients with MCI showed lower cognitive functions, serum high-density lipoprotein (HDL-c), and apolipoprotein A1 (apoA-I) levels; and higher total cholesterol level than the controls. Serum HDL-c (*P* = 0.001) and apoA-I (*P* = 0.016) were positively associated with the MoCA score in MCI patients. Further stratification analyses revealed that the subjects with higher HDL-c concentration showed better attention and memory for verbal, visual, and logical functions than the group with lower HDL-c concentration (*P* < 0.05). No significant differences were observed among the distributions of ABCA1 R219K variants between MCI patients and controls; however, the KK genotype carriers presented higher apoA-I levels than those with RR genotype in MCI individuals.

**Conclusion:** This study does not support the association between R219K polymorphism and T2DM-related MCI. However, our data suggested that the serum HDL-c level might positively influence cognition, especially memory function, in T2DM patients. Further studies are needed to determine the interaction between lipids and ABCA1 genotype and its effect on cognition in T2DM patients.

**Trial registration:** Advanced Glycation End Products Induced Cognitive Impairment in Diabetes: BDNF Signal Meditated Hippocampal Neurogenesis ChiCTR-OCC-15006060; http://www.chictr.org.cn/showproj.aspx?proj=10536.

## Introduction

Alzheimer's disease (AD) and type 2 diabetes mellitus (T2DM) are both age related diseases, characterized by elevated incidence with aging (Kukull et al., 2002; Ahmad, 2013). T2DM individuals have higher risk for AD than non-diabetic individuals (Peila et al., 2002; Ahtiluoto et al., 2010). Mild cognitive impairment (MCI) is an interim stage between normal cognition aging and dementia and accounts for an increased risk for AD (Petersen et al., 1999). Studies consistently suggested a positive association between T2DM and MCI and an advanced development from MCI to AD in T2DM (Luchsinger et al., 2007; Alagiakrishnan and Sclater, 2012).

Despite not being clearly understood, the possible association between cognitive impairment and plasma lipids in T2DM has gained increasing attention (Chen et al., 2011; Ahmad, 2013). The increased levels of triglycerides (TG), total cholesterol (TC), and low-density lipoprotein cholesterol (LDL-c), as well as the decreased levels of high-density lipoprotein cholesterol (HDL-c), are risk factors for T2DM and neurodegenerative disorders, such as AD (Jenkins et al., 2004; Eckel et al., 2005; Lutjohann, 2006; Shepherd, 2007; Hayashi, 2011). High levels of plasma HDL-c are associated with a significantly reduced risk for AD (Vollbach et al., 2005; Reitz et al., 2010), whereas low HDL-c levels are associated with low hippocampal volume (Wolf et al., 2004) and greatly related with AD severity (Merched et al., 2000). In addition, HDL-c is positively associated with verbal memory performance (Singh-Manoux et al., 2008) and is a strong predictor of future development of AD or dementia (Elias et al., 2000); however, the exact mechanism of plasma lipids and lipoproteins on cognition remains conflicting (van Exel et al., 2002; Yaffe et al., 2002; Sabbagh et al., 2004; Anstey et al., 2008). Besides that, the relationship between plasma lipids and T2DM associated MCI is limited, especially in different cognitive domains.

Cholesterol metabolism plays a potential role in the pathology of cognitive impairment in T2DM (Ahmad, 2013) and the formation of the amyloid-β (Aβ), which is an important pathophysiological characteristic of AD (Hardy and Selkoe, 2002); therefore, the genes contributing to the variance in both lipid processing and Aβ formation are promising as biological targets for studying MCI in T2DM. In addition to apolipoprotein E (APOE), the gene encoding the ATP-binding cassette A1 transporter (ABCA1) has received increasing attention. Located on the long arm of chromosome 9, the ABCA1 gene regulates the transport of cellular cholesterol and phospholipids from the inner to the outer layer through the cell membrane and is important for the synthesis of HDL and lipoproteins (Oram and Lawn, 2001). Moreover, the ABCA1 variant is associated with T2DM onset (Haghvirdizadeh et al., 2015). The crucial role of ABCA1 in pancreatic β-cell function has been observed in a mouse model (Brunham et al., 2007). A weak link between ABCA1 and the physiopathology of AD has been identified in recent gene association studies (Katzov et al., 2004; Rodriguez-Rodriguez et al., 2007). One of the most convincing ABCA1 polymorphisms is R219K (rs2230806). ABCA1 has a potential role in the progress of cognitive impairment; a deficiency in ABCA1 reduces the brain APOE but increases the Aβ levels (Hirsch-Reinshagen et al., 2005; Koldamova et al., 2005). Moreover, the K allele of the R219K polymorphism in ABCA1 gene is associated with the increased plasma HDL-c and decreased TG levels (Clee et al., 2001; Miller et al., 2003), which might be related with the late-onset AD (Kehoe et al., 1999; Burns et al., 2006; Donkin et al., 2010). Although the relationship between ABCA1 and AD has been widely investigated, how the ABCA1 R219K variant affects diabetes-related MCI has not been reported.

We hypothesized that the adverse lipid profiles and the polymorphism of ABCA1, a HDL-c related gene, might be crucial in modulating the pathogenesis of cognitive impairment in diabetic patients. Therefore, the present study aimed to analyze the influence of ABCA1 R219K polymorphism on lipid profiles and T2DM-associated MCI, and investigate the relationships between plasma lipids and multiple cognitive domains of MCI in T2DM patients. Our findings will provide additional insights into the cholesterol pathogenesis in cognitive impairments in T2DM.

## Materials and methods

### Study subjects

A total of 226 T2DM patients who were hospitalized at the Department of Endocrinology of the Affiliated Zhongda Hospital of Southeast University from May to October 2016 were enrolled in this study. Written informed consent was obtained from all patients prior to their participation in the study. The study protocol was approved by the Research Ethics Committee of the Affiliated Zhongda Hospital of Southeast University.

A brief screening for enrollment was conducted to identify the potential participants. We included right-handed patients aged 40–80 years with T2DM for over 3 years. We excluded patients according to the following criteria: (1) with history with visual or hearing loss that render them unable to cooperate with the study procedure; (2) with history of head injury, epilepsy, Parkinson's disease, known neurological degenerative diseases, or cerebrovascular accidents as confirmed by neuroimaging scans within 1 year, and other mental illnesses; (3) with history of hypoglycemic coma, diabetic ketoacidosis, lactic acidosis, hyperosmolar non-ketotic diabetic coma, or other acute diabetic complications for the past 3 months; (4) with major medical illnesses, such as cancer, serious infection, chronic renal failure, heart failure, lung disease, and thyroid disease; (5) with history of drug or alcohol abuse within 2 months; and (6) with history of depression within 2 months or history of antidepressant or other cognition-impairment drug use in the past 3 months. The exclusion criteria in this study were applied to both the MCI and healthy cognition controls. According to the 2006 European Alzheimer's Disease Consortium criteria (Portet et al., 2006) and the Montreal Cognitive Assessment (MoCA) scoring system (Nasreddine et al., 2005), we recruited 124 MCI patients and 102 patients with healthy cognition.

### Clinical assessment

The baseline characteristics of age (years), gender (male or female), educational level (years), diabetes duration (years), smoking habits (yes or no), alcohol consumption (yes or no), body mass index (BMI), history of hypertension (yes or no), systolic blood pressure, and diastolic blood pressure were collected. Fasting blood glucose (FBG) and glycosylated hemoglobin (HbA1c) were measured using standard laboratory techniques.

### Lipid/lipoprotein measurement

Blood samples were collected to measure the serum lipid/lipoprotein. Serum TC, TG, LDL-c, HDL-c, apolipoprotein A1 (apoA-I), and apolipoprotein B (ApoB) were measured using standard laboratory techniques in the central laboratory of the Zhongda Hospital as directed by the Chinese Laboratory Quality Control. According to American Diabetes Association Complete Guide to Diabetes (2015) and China Guideline for Type 2 Diabetes (Ju-Ming et al., 2014), the lipid parameters of individuals were stratified as follows: appropriate TC level (<4.5 mmol/L) or higher TC (≥4.5 mmol/L); appropriate TG level (<1.7 mmol/L) or higher TG (≥1.7 mmol/L); appropriate LDL-c level (<2.6 mmol/L) or higher LDL-c (≥2.6 mmol/L); appropriate HDL-c level (>1.0 mmol/L) or lower HDL-c (≤ 1.0 mmol/L) for men; and appropriate HDL-c level (>1.3 mmol/L) or lower HDL-c (≤ 1.3 mmol/L) for women.

### Cognitive function assessment

All participates underwent MoCA, Digit Span Test, Trail Making Test-A, Trail Making Test-B, Auditory Verbal Learning Test, Complex Figure Test, and Logic Memory Test to evaluate their psychomotor speed, executive function, attention, verbal and visual functional memory, and logical memory. MoCA is used to measure global cognition with a cut point ≥26 (one point added if the individual has fewer than 12 years of education), which is defined as normal cognition. Hamilton Depression Rating Scale, Self-rating Depression scale, Activity of Daily Living Scale, Hachinski Ischemic Scale, and Clinical Dementia Rating Scale were also used for assessment.

### Genotyping

Blood samples were used to extract DNA according to the manufacturer's instructions (Puregene, GentraSystem, Minneapolis, MN). Variants of ABCA1 gene (the R219K polymorphism) were examined through polymerase chain reaction-restriction fragment length polymorphism using the primers 5′-GATGGCCCAAAAGTCTGAAA-3′ (forward) and 5′-GGACTGTTGCAATGGAACCT-3′ (reverse). PCR was conducted in a 30 μL reaction mixture containing 3.0 μL of 10 × PCR buffer, 60 ng of DNA, 2 μL of dNTP, 10 pmol of forward primer, 10 pmol of reverse primer, and 20.8 μL of ddH_2_O. The amplification was initiated for preliminary denaturation at 96°C for 5 min, 30 cycles of denaturation at 96°C for 20 s, annealing at 62°C for 20 s, extension at 72°C for 30 s, and another extension at 72°C for 10 min. The detection of was performed on 8–2.5% agarose gel electrophoresis for 30 min. Three fragments were generated after digestion with EcoNI.

### Statistical analysis

Statistical analyses were conducted using SPSS 20.0. Data were presented as median (interquartile range), mean ± standard deviation, or percentages. Student's *t*-test and one-way ANOVA were used for normally distributed continuous variables, whereas non-parametric Mann-Whitney *U* or Kruskal-Wallis test was used for asymmetrically distributed continuous variables. Qualitative variables and the Hardy-Weinberg equilibrium of the allelic and genotypic distributions were compared using the chi-square (χ2) test. Pearson or Spearman rank correlation analysis for normally or non-normally distributed variables was used to evaluate the relationships.

## Results

### Baseline characteristics, serum lipid levels, and cognitive performance

Among the included 226 T2DM patients, 124 were MCI, and 102 were controls with healthy cognition. The age and gender of MCI and healthy cognition groups matched well. The two groups showed no significant differences in smoking habits, alcohol consumption, diabetes duration, BMI, hypertension percentages, diastolic blood pressure, and HbA1c, FBG, TG, LDL-c, and apoB levels (*P* > 0.05). The education years (*P* < 0.001), HDL-c (*P* = 0.003) and apoA-I (*P* = 0.021) serum levels in the MCI group were lower than those in the control group. Although both education years and HDL-c level were significantly different between the two groups, they were not correlated (*r* = 0.078, *P* = 0.248). After correcting for years of education years, the difference in HDL-c level between MCI and control group was still statistically significant (*P* = 0.017). What's more, the percentages of history of hyperlipidemia, systolic blood pressure, and serum TC level were higher in the MCI group than in the healthy cognition group (*P* < 0.05). The MCI group exhibited significantly worse cognitive performance on psychomotor speed, executive function, attention, verbal memory, visual memory, and logical memory than the control group (*P* < 0.05) (Table 1).

**Table 1:** Comparison of baseline characteristics between MCI patients and controls.

**Characteristic**	**MCI group (*n* = 124)**	**Control group (*n* = 102)**	***P***
Age (years)	62.46 ± 8.44	60.52 ± 7.98	0.079
Female, *n* (%)	63 (50.81)	46 (45.10)	0.393
Education levels (years)	8.30 ± 4.09	10.95 ± 3.46	< 0.001
Ever smoked, *n* (%)	60 (48.39)	41 (40.20)	0.218
Alcohol consumption, *n* (%)	38 (30.65)	36 (35.29)	0.459
Diabetes duration (years)	10.35 ± 6.12	9.06 ± 4.89	0.085
Hyperlipidemia (%)	94 (75.81)	64 (62.75)	0.033
BMI (kg/m^2^)	25.08 ± 3.58	25.03 ± 3.34	0.909
Hypertension, *n* (%)	75 (60.48)	59 (57.84)	0.688
SBP (mmHg)	138.53 ± 18.60	133.66 ± 14.21	0.027
DBP (mmHg)	82.42 ± 10.94	81.40 ± 10.01	0.471
HbA1c (%)	9.54 ± 2.22	9.03 ± 2.34	0.105
FBG(mmol/L)	8.08 ± 2.73	7.66 ± 2.59	0.252
TG (mmol/L)	2.15 ± 1.99	2.08 ± 1.56	0.786
TC (mmol/L)	5.11 ± 1.55	4.69 ± 1.25	0.032
HDL-c (mmol/L)	1.20 ± 0.31	1.31 ± 0.25	0.003
LDL-c (mmol/L)	3.11 ± 0.88	2.98 ± 0.88	0.289
apoA-I (g/L)	1.07 ± 0.23	1.16 ± 0.26	0.021
ApoB (g/L)	0.82 (0.70–0.92)	0.85 (0.69–1.02)	0.373
**COGNITION TEST LEVELS**			
Global cognition	21.00 (18.00–23.00)	27.00 (26.00–28.00)	< 0.05
Psychomotor speed	81.00 (60.00–109.75)	58.00 (47.75–71.00)	< 0.05
Attention	10.50 (8.00–12.00)	12.00 (11.00–14.00)	< 0.05
Executive function	197.00 (150.00–273.25)	142.50 (112.25–179.25)	< 0.05
Verbal memory	4.14 ± 2.39	7.13 ± 2.75	< 0.05
Logical memory	5.04 ± 3.59	8.70 ± 3.61	< 0.05
Visual memory	8.00 (7.00–10.00)	16.00 (13.00–19.00)	< 0.05

*Data are presented as n (%), mean ± SD, or median (interquartile range) as appropriate. Student's t-test, Mann-Whitney U-test, or × ^2^ test were used for comparison variables, MCI, mild cognitive impairment; BMI, body mass index; SBP, systolic blood pressure; DBP, diastolic blood pressure; HbA1c, glycosylated hemoglobin; FBG, fasting blood-glucose; TG, triglyceride; TC, total cholesterol; HDL-c, high density lipoprotein cholesterol; LDL-c, low density lipoprotein cholesterol; apoA-I: apolipoprotein A1; ApoB: apolipoprotein B*.

### Correlations between global cognition (MoCA score) and clinical variables

The correlations between global cognition (MoCA score) and clinical variables are presented in Table 2. TG, TC, LDL-c, and ApoB levels were not significantly correlated with the MoCA score whether in all subjects or MCI individuals (*P* > 0.05). After adjusting for age, the education years (*r* = 0.474, *P* < 0.001; *r* = 0.471, *P* < 0.001), and levels of HDL-c (*r* = 0.143, *P* = 0.043; *r* = 0.301, *P* = 0.001), and apoA-I (*r* = 0.146, *P* = 0.039; *r* = 0.230, *P* = 0.016) were positively correlated with MoCA score, whereas the age was inversely associated with MoCA score (*r* = −0.253, *P* < 0.001; *r* = −0.208, *P* = 0.020) in all groups and MCI group, separately (Table 2). The correlation of the MoCA score with other clinical indicators were not examined in the controls individually because the interval of the MoCA score in the controls was narrow.

**Table 2:** Correlation of cognitive function with clinical indicators in T2DM subjects.

	**MoCA score**							
	**All groups**				**MCI group**			
	***R***	***P*^a^**	***R***	***P*^b^**	***R***	***P*^a^**	***R***	***P*^b^**
Age (years)	−0.253	<0.001			−0.208	0.020		
Education levels (years)	0.427	<0.001	0.474	<0.001	0.482	<0.001	0.471	<0.001
TG (mmol/L)	−0.028	0.669	−0.011	0.876	−0.019	0.837	0.056	0.563
TC (mmol/L)	−0.043	0.523	−0.030	0.672	−0.045	0.622	0.025	0.797
LDL- c (mmol/L)	−0.044	0.513	−0.034	0.738	−0.015	0.869	−0.028	0.775
HDL- c (mmol/L)	0.078	0.241	0.143	0.043	0.203	0.026	0.301	0.001
apoA-I (g/L)	0.133	0.059	0.146	0.039	0.245	0.010	0.230	0.016
ApoB (g/L)	0.056	0.429	0.062	0.380	−0.034	0.721	−0.032	0.740

a *Spearman rank correlation analysis for abnormally distributed variables*.

b *Partial correlation analysis adjusted for age*.

*T2DM, type 2 diabetes mellitus; MCI, mild cognitive impairment; MoCA, Montreal Cognitive Assessment; TG, triglyceride; TC, total cholesterol; LDL-c, low density lipoprotein cholesterol; HDL-c, high density lipoprotein cholesterol; apoA-I: apolipoprotein A1; ApoB: apolipoprotein B*.

### Comparison of different cognitive domains between different serum lipid levels

Table 3 shows the analysis of the interactions between serum lipids and cognitive performances. We split serum lipids according to blood lipid management targets in diabetes in strict accordance with American Diabetes Association Complete Guide to Diabetes (2015) and China Guideline for Type 2 Diabetes (Ju-Ming et al., 2014). The results showed that individuals with high HDL-c concentration (>1.0 mmol/L for men and >1.3 mmol/L for woman) had better global cognition and higher scores of attention, verbal memory, logical memory, and visual memory than those with lower HDL-c (HDL-c ≤ 1.0 mmol/L for men and ≤ 1.3 mmol/L for women) (*P* < 0.05). No association was found between the psychomotor speed, executive function, and serum level of HDL-c (*P* > 0.05). Subjects with TC levels of <4.5 mmol/L had higher global cognition and verbal memory scores (*P* = 0.028) than patients with TC levels of ≥4.5 mmol/L; however, the differences disappeared after adjusting for education levels. The two groups showed no differences in the scores of attention, psychomotor speed, executive function, logical memory, and visual memory (*P* > 0.05). TG and LDL-c concentrations had no influence on the cognitive scores (*P* > 0.05). Similar results were obtained when adjusted for education levels (Table 3).

**Table 3:** Comparison of cognitive outcomes between different lipid levels in all T2DM patients.

**Cognitive tests**	**Stratified by different lipid levels**		***P***	***P*^a^**
	**HDL-c category**			
	**HDL > 1.0 or 1.3 mmol/L**	**HDL ≤ 1.0 or 1.3 mmol/L**		
Global cognition	25.00 (22.00–27.00)	22.00 (19.00–25.50)	<0.001	<0.001
Psychomotor speed	65.00 (51.00–87.00)	70.00 (53.50–96.50)	0.102	0.159
Executive function	160.00 (130.00–210.00)	177.00 (130.00–256.50)	0.164	0.142
Verbal memory	5.82 ± 3.14	4.90 ± 2.52	0.018	0.047
Logical memory	7.24 ± 4.20	5.86 ± 3.55	0.014	0.030
Visual memory	12.00 (8.00–16.00)	10.00 (7.50–12.00)	0.006	0.018
Attention and working memory	11.00 (10.00–13.00)	11.00 (9.00–12.50)	0.017	0.023
	**TG category**			
	**TG < 1.7 mmol/L**	**TG ≥ 1.7 mmol/L**		
Global cognition	25.00 (21.00–27.00)	24.00 (20.00–26.00)	0.280	0.193
Psychomotor speed	65.00 (51.00–85.00)	70.00 (54.00–99.50)	0.133	0.704
Executive function	155.00 (127.00–206.00)	178.00 (140.50–234.25)	0.066	0.359
Verbal memory	5.46 ± 2.72	5.56 ± 3.20	0.810	0.722
Logical memory	6.62 ± 4.02	6.89 ± 4.03	0.615	0.542
Visual memory	11.00 (8.00–16.00)	11.00 (8.00–14.75)	0.839	0.690
Attention and working memory	11.00 (10.00–13.00)	11.00 (9.00–13.00)	0.496	0.841
	**LDL-c category**			
	**LDL < 2.6 mmol/L**	**LDL ≥ 2.6 mmol/L**		
Global cognition	25.00 (21.00–27.00)	24.00 (21.00–27.00)	0.230	0.312
Psychomotor speed	68.00 (53.25–91.50)	67.00 (52.00–91.50)	0.754	0.715
Executive function	167.50 (134.00–231.75)	167.00 (128.00–220.00)	0.737	0.565
Verbal memory	5.74 ± 3.06	5.41 ± 2.89	0.440	0.490
Logical memory	6.85 ± 4.25	6.77 ± 3.91	0.887	0.955
Visual memory	12.00 (8.00–17.00)	10.00 (8.00–14.00)	0.146	0.129
Attention and working memory	11.00 (9.25–13.00)	11.00 (10.00–13.00)	0.757	0.589
	**TC category**			
	**TC < 4.5 mmol/L**	**TC ≥ 4.5 mmol/L**		
Global cognition	25.00 (21.50–27.00)	24.00 (20.00–26.00)	0.028	0.263
Psychomotor speed	65.00 (49.00–90.00)	69.00 (53.00–94.50)	0.306	0.494
Executive function	160.00 (128.50–214.50)	168.00 (130.00–229.50)	0.377	0.930
Verbal memory	6.02 ± 2.95	5.17 ± 2.89	0.033	0.080
Logical memory	6.97 ± 3.94	6.64 ± 4.07	0.544	0.817
Visual memory	11.00 (8.00–16.00)	11.00 (8.00–15.00)	0.568	0.760
Attention and working memory	11.00 (10.00–13.00)	11.00 (9.00–12.50)	0.249	0.484

a *Adjusted for education levels*.

*Data are presented as mean ± SD or median (interquartile range) as appropriate*.

*Student's t-test or Mann-Whitney U-test were used for comparison variables*.

*T2DM, type 2 diabetes mellitus; HDL-c, high density lipoprotein cholesterol; TG, triglyceride; LDL-c, low density lipoprotein cholesterol; TC, total cholesterol*.

### Distributions of ABCA1 polymorphisms in MCI and non-MCI groups

The R219K polymorphism distributions followed the Hardy-Weinberg equilibrium in both MCI cases (χ^2^ = 0.04, *df* = 1, *P* > 0.05) and controls (χ^2^ = 0, *df* = 1, *P* > 0.05). The MCI and control groups showed no difference in ABCA1 (R219K) genotype (χ^2^ = 0.697, *df* = 2, *P* = 0.706) and allele distribution (χ^2^ = 0.679, *df* = 1, *P* = 0.410). After adjusting for age, gender, and education years, no interactions were found between the distributions of R219K polymorphism and cognitive function (Table 4).

**Table 4:** Distributions of ABCA1(R219K) genotype and allele frequencies in MCI and normal control with T2DM.

**Genotype and alleles**	**MCI, *n* (%)**	**Non-MCI, *n* (%)**	**Crude OR (95% CI)**	***P***	**Adjusted OR (95% CI)^a^**	***P***
Overall	124	102				
R	140 (56.45)	123 (60.29)	1.000			
K	108 (43.55)	81 (39.71)	1.171 (0.804–1.707)	0.410		
RR	39 (31.45)	37 (36.27)	1.000		1.000	
RK	62 (50.00)	49 (48.04)	1.200 (0.669–2.156)	0.541	1.172 (0.626–2.196)	0.620
KK	23 (18.55)	16 (15.69)	1.364 (0.625–2.977)	0.435	1.103 (0.730–1.666)	0.643
RK + KK	85 (68.55)	65 (63.73)	1.241 (0.713–2.158)	0.445	1.081 (0.806–1.449)	0.605

*Data are presented as n (%)*.

*×^2^ test was used to compare the genotype and allele frequencies*.

a *Adjusted for age, education level, sex*.

*ABCA1, ATP-binding cassette transporter A1; MCI, mild cognitive impairment; T2DM, Type 2 diabetic mellitus*.

### ABCA1 R219K polymorphisms and serum lipid levels

No significant differences in the blood lipid levels were found among R219K SNP in all subjects. No associations between ABCA1 R219K gene polymorphisms and serum lipid levels were found in control group. However, in the MCI group, a significant difference in apoA-I distribution was detected according to the R219K genotype (*P* = 0.021). The apoA-I level increased in ascending order during RR, RK, KK genotypes. In addition, the increased tendency of HDL-c level was also displayed in ascending order according to RR, RK, KK genotypes; however, the change was not statistically significant (*P* = 0.055). The TG, TC, LDL-c, and ApoB levels did not vary among the different ABCA1 genotypes in MCI patients (Table 5).

**Table 5:** Comparison of lipid levels according to ABCA1 R219K polymorphism.

**Parameters**	**All group (*n* = 226)**			***P***	**MCI group (*n* = 124)**			***P***	**Control group(*n* = 102)**			***P***
	**RR**	**RK**	**KK**		**RR**	**RK**	**KK**		**RR**	**RK**	**KK**	
TG(mmol/L)	2.08 ± 1.44	2.02 ± 2.01	2.46 ± 1.84	0.426	2.05 ± 1.32	2.02 ± 2.28	2.49 ± 2.13	0.367	2.12 ± 1.57	2.03 ± 1.64	2.14 ± 1.35	0.953
TC(mmol/L)	4.94 ± 1.23	4.89 ± 1.59	4.94 ± 1.35	0.968	5.25 ± 1.36	5.05 ± 1.71	5.01 ± 1.42	0.782	4.61 ± 0.99	4.70 ± 1.41	4.86 ± 1.30	0.809
HDL(mmol/L)	1.19 ± 0.25	1.27 ± 0.31	1.30 ± 0.29	0.109	1.11 ± 0.25	1.22 ± 0.34	1.29 ± 0.26	0.055	1.29 ± 0.22	1.33 ± 0.24	1.31 ± 0.33	0.790
LDL(mmol/L)	3.11 ± 0.83	3.02 ± 0.96	3.01 ± 0.74	0.781	3.29 ± 0.89	3.03 ± 0.92	2.99 ± 0.71	0.276	2.91 ± 0.71	3.01 ± 1.01	3.04 ± 0.80	0.817
apoA-I (g/L)	1.06 ± 0.23	1.13 ± 0.26	1.16 ± 0.23	0.093	0.99 ± 0.17	1.11 ± 0.25	1.14 ± 0.24	0.021	1.14 ± 0.26	1.16 ± 0.26	1.17 ± 0.26	0.896
ApoB (g/L)	0.81(0.73–0.97)	0.82(0.67–0.95)	0.84(0.71–0.92)	0.921	0.80(0.72–1.01)	0.82(0.67–0.92)	0.80(0.70–0.92)	0.878	0.81(0.74–0.96)	0.86(0.65–1.05)	0.87(0.76–1.02)	0.891

*Data are presented as mean ± SD or median (interquartile range) as appropriate*.

*One-way ANOVA for the comparison of the lipid levels between different genotypes of ABCA1*.

*ABCA1, ATP-binding cassette transporter A1; MCI: mild cognitive impairment; TG, triglyceride; TC, total cholesterol; HDL-c, high density lipoprotein cholesterol; LDL-c, low density lipoprotein cholesterol; apoA-I: apolipoprotein A1; ApoB: apolipoprotein B*.

## Discussion

This study contributed in the understanding of the associations between cholesterol, genetics, and cognitive function in T2DM. T2DM patients with MCI had significantly lower HDL-c and apoA-I levels and higher TC level than T2DM patients with healthy cognition. The serum levels of HDL-c and apoA-I were positively correlated with cognitive function. The increased HDL-c level was associated with improved memory. However, no association between ABCA1 rs2230806 polymorphism and MCI was found in T2DM patients, although an increased tendency of the HDL-c level and apoA-I concentration were observed in the KK genotype.

Several observational studies have investigated the relationship between plasma HDL-c level and cognitive function; however, the results are conflicting(Merched et al., 2000; Launer et al., 2001; Atzmon et al., 2002; Reitz et al., 2004, 2008; Li et al., 2005). In this study, the HDL-c level in T2DM with MCI was significantly lower than that in the controls; the elevated serum HDL-c level was associated with better cognitive and memory functions but not psychomotor speed and executive function. These findings are consistent with previous studies, which suggested that plasma HDL-c has a positive role in cognitive function (Merched et al., 2000; Atzmon et al., 2002), and that low HDL-c level is associated with poor memory (Singh-Manoux et al., 2008). Conversely, other scholars have different opinions. Li et al. stated that low HDL levels might be related to a low risk of cognitive impairment (Li et al., 2005). The Honolulu Asia Aging Study also suggested that increased HDL-c level was associated with elevated neuritic plaques and neurofibrillary tangles in both mid and late-life stages (Launer et al., 2001). These different results might be explained by the following reasons. First, the ethnic differences might have led to the differences in these studies. Second, differences in population characteristics appeared to be notable reasons. This study, included only the participants with at least 3 years of T2DM. T2DM is commonly characterized by dyslipidemia with reduced serum HDL-c levels compared with non-diabetic individuals (Shepherd, 2007). However, only a few studies included T2DM patients. In the current study, the diabetic status resulted in profound changes in lipid levels, which possibly contributed to the association of MCI and HDL-c. Moreover, the measurement error of cognitive domains or differences in measurement methods according to different cultural background might also cause different results. In the current study, the reduced plasma apoA-I was observed in MCI patients and was correlated with significant deficits in cognitive function. This finding is consistent with the association of serum apoA-I concentrations and AD severity (Merched et al., 2000). Laboratory studies indicated that in APP/PS1 mouse models of AD, the genetic overexpression of apoA-I prevented the development of cognitive impairment, whereas the deficiency of apoA-I exacerbated cognitive impairments (Lewis et al., 2010). With regard to serum TC, we only observed high levels of serum TC in MCI, but no correlation was found between TC and MoCA scores. However, this finding does not necessarily dismiss the role of TC in the pathology of cognitive deficits because serum TC is not equal to the cholesterol level in the brain (Bjorkhem and Meaney, 2004). No association was found among serum TG, LDL-c, and ApoB levels and cognitive function in the current study. Notably, there's significantly difference of years of education on the cognition between MCI and controls. This is consistent with other studies suggested that higher education is associated with reduced risk of dementia (Qiu et al., 2001; Anttila et al., 2002). Studies have suggested individuals with higher education may be exposed to less toxins and have healthier lifestyles that lower the likelihood of brain injury (Del Ser et al., 1999). Besides this, individuals with higher education may also be better able to adjust to the pathological changes of dementia and reserve cognitive function (Stern et al., 1994). Although evidence confirmed the protective role of plasma HDL-c on cognition, the underlying mechanisms are poorly understood. One plausible explanation is that the HDL-c level is related to small-vessel diseases through the effect on the removal of excess cholesterol from subendothelial spaces of cerebral microvessels (Zuliani et al., 2001). Low HDL-c concentrations are associated with atherosclerotic diseases (Sharrett et al., 1994) and result in ischemic lesions in the brain and thus lead to cognitive impairment (Breteler et al., 1994). Second, HDL-c might be involved in preventing the aggregation and polymerization of amyloid proteins in the brain (Koudinov et al., 1998; Olesen and Dago, 2000). This phenomenon might slow down the development of cognitive impairment. What's more, the anti-inflammatory (Cockerill et al., 2001) and antioxidative (Paterno et al., 2004) properties of HDL-c might play an important role in protecting cognition. In line with this, many studies have suggested that drugs with anti-inflammatory properties might protect individuals from cognitive decline (Breitner et al., 1994; Wolozin et al., 2000; Albert et al., 2001; in t' Veld et al., 2001). Furthermore, higher apoA-I and HDL-c levels are associated with the formation of Apo J complexes, which can affect the blood-brain barrier transport and maintain the function of intact brain cells (Urist, 1975).

Recently, a few studies suggested the important role of ABCA1 on the pathology of AD (Koldamova et al., 2005; Wahrle et al., 2008; Elali and Rivest, 2013). However, the role of the R219K polymorphism of the ABCA1 gene in AD patients remains controversial (Katzov et al., 2004; Li et al., 2004; Rodriguez-Rodriguez et al., 2007). Consistent with previous studies (Li et al., 2004) (Kolsch et al., 2006; Shibata et al., 2006; Wahrle et al., 2007), we failed to detect the association of the ABCA1 R291K genotype distributions and allele frequencies between MCI and controls of T2DM patients. Differ from our results, there's evidence showed that the RK + KK in R219K (Rodriguez-Rodriguez et al., 2007; Sun et al., 2012) and 219K allele (Chu et al., 2007) were associated with high risk of AD. In contrast, Katzov et al. (2004) and Wang and Jia (2007) found a significantly higher frequency of the R219K/KK genotype among the controls compared with AD. Several discrepancies might explain these inconsistent findings. First, population-dependent differences might be an important reason cause the different results because ABCA1 polymorphism distributions were different between different ethnicities. Second, a small sample size might increase the probability of false associations between genetic polymorphism and cognitive decline. Moreover, environmental factors, such as lifestyle and obesity might influence the progress of cognitive impairment and thus lead to discrepancies (Mody et al., 2011). Further analysis about the ABCA1 R219K variant with plasma lipids in the current study showed that in MCI patients, the apoA-I level increased in ascending order during RR, RK, KK genotypes with statistical difference between the RR and KK genotypes. The increased tendency of HDL-c level was also displayed in ascending order according to RR, RK, KK genotypes, but no statistically significance was observed. This finding is consistent with the meta-analysis, which suggested a significantly higher level of HDL-c in the carriers of KK genotype and K allele in Asian populations (Ma et al., 2011); and the KK genotype and K allele are associated with higher plasma HDL-c concentration in AD patients (Xiao et al., 2012). These findings partly reflect the interactions between ABCA1 with lipids (HDL-c and apoA-I) as mediated by R219K (an N-terminal extracellular loop) (Fitzgerald et al., 2002). The underlying mechanism for the different associations between ABCA1 R219K polymorphism and cognitive decline remains unclear. ABCA1 219K allele has been suggested to increase the ABCA1 protein function by accelerating cholesterol efflux, altering Aβ secretion, and influencing APOE metabolism, and any of which might be critical in the etiology of cognitive impairment (Wang and Jia, 2007). However, another study pointed out that the subjects with the 219K allele have significantly higher CSF A42 levels than those with RR homozygotes (Katzov et al., 2006). Further investigation should be conducted to explore the mechanism of ABCA1 gene variation in cognitive deficiencies.

Our study exhibits several strengths. This study presents insights into plasma lipids and ABCA1 R219K polymorphism underlying the cognition status in T2DM subjects and measures different cognitive domains including different memory functions. We used cholesterol cut-offs according to ADA Complete Guide to Diabetes and China Guideline for Type 2 Diabetes. Hence, the findings in our study are practical. However, the following limitations should be noted. First, this study used only one measurement of lipid concentrations, which might have resulted in measurement errors and misestimated associations between lipids and cognitive impairments. Second, the present study is not a follow-up study; thus, we cannot measure the longitudinal levels of lipids during aging. Third, the relatively small sample size renders it difficult to consider APOE polymorphism and investigate the genotype-genotype associations. Fourth, due to hospitalization, some patients might not being at their baseline characteristics, which might have disproportionately affected cognitive performance. This feature is an important confounder that influences cognitive status evaluation and thus affected the results of this study. Finally, the pathogenic mechanism of diabetes-related cognitive impairment is complex and related to many factors. Thus, the contribution of this study to scientific fields is limited, and further investigations are needed to discover diabetes-related cognitive dysfunctions.

## Conclusion

In conclusion, our findings showed that serum HDL-c level positively affects cognitive functions, especially memory function in T2DM. Although the polymorphism of ABCA1 R219K and the risk of cognitive impairment in T2DM patients are not associated, the ABCA1 KK genotype might contribute to the high serum apoA-I level. Combined with the clinical characteristics of T2DM patients, therapeutic strategies that enhance serum HDL-c might prevent the development of cognitive decline in diabetic patients. However, comprehensive studies with large sample sizes and involving different ethnic populations are required to understand the contribution of the ABCA1 genotype to dyslipidemia and its further influence on cognition in T2DM.

## Author contributions

SW contributed to the design of the manuscript. RC contributed to the idea of the manuscript, conducted of the study and wrote the manuscript. JH carried out the data collection. JS revised the manuscript. RH participated in the data analysis. YS and ST helped data interpretation. All authors read and approved the final manuscript.

### Conflict of interest statement

The authors declare that the research was conducted in the absence of any commercial or financial relationships that could be construed as a potential conflict of interest.
